# Preliminary study: quantification of chronic pain from physiological data

**DOI:** 10.1097/PR9.0000000000001039

**Published:** 2022-10-04

**Authors:** Zhuowei Cheng, Franklin Ly, Tyler Santander, Elyes Turki, Yun Zhao, Jamie Yoo, Kian Lonergan, Jordan Gray, Christopher H. Li, Henry Yang, Michael Miller, Paul Hansma, Linda Petzold

**Affiliations:** Departments of a Computer Science,; b Mechanical Engineering,; c Psychological & Brain Sciences, and; d Physics, University of California, Santa Barbara, Santa Barbara, CA, USA

**Keywords:** Chronic pain, Physiological data, Pain quantification, Random forest

## Abstract

**Introduction:**

It is unknown if physiological changes associated with chronic pain could be measured with inexpensive physiological sensors. Recently, acute pain and laboratory-induced pain have been quantified with physiological sensors.

**Objectives:**

To investigate the extent to which chronic pain can be quantified with physiological sensors.

**Methods:**

Data were collected from chronic pain sufferers who subjectively rated their pain on a 0 to 10 visual analogue scale, using our recently developed pain meter. Physiological variables, including pulse, temperature, and motion signals, were measured at head, neck, wrist, and finger with multiple sensors. To quantify pain, features were first extracted from 10-second windows. Linear models with recursive feature elimination were fit for each subject. A random forest regression model was used for pain score prediction for the population-level model.

**Results:**

Predictive performance was assessed using leave-one-recording-out cross-validation and nonparametric permutation testing. For individual-level models, 5 of 12 subjects yielded intraclass correlation coefficients between actual and predicted pain scores of 0.46 to 0.75. For the population-level model, the random forest method yielded an intraclass correlation coefficient of 0.58. Bland–Altman analysis shows that our model tends to overestimate the lower end of the pain scores and underestimate the higher end.

**Conclusion:**

This is the first demonstration that physiological data can be correlated with chronic pain, both for individuals and populations. Further research and more extensive data will be required to assess whether this approach could be used as a “chronic pain meter” to assess the level of chronic pain in patients.

## Abstract 2

Supplemental Digital Content is Available in the Text.

Preliminary evidence suggests that physiological variables collected with our low-cost pain meter are correlated with chronic pain, both for individuals and populations.

## 1. Introduction

Chronic pain is a significant public health problem. Recent estimates show that 50 million U.S. adults had chronic pain.^22^ Chronic pain and pain-related disability in the United States costs $560 to $650 billion dollars, far exceeding the costs of cardiovascular disease, cancer, and diabetes.^36^ Yet, despite the high cost and the profound social and personal burdens imposed by chronic pain, the clinical means by which we quantify levels of pain are largely relied on subjective self-reports rather than objective measures of pain intensity.^18^

A large body of work demonstrates that chronic pain causes changes in the brain that cause pain to persist beyond tissue healing. In particular, functional magnetic resonance imaging (fMRI) has been used to demonstrate changes in the brain that are consistent with chronic pain.^3–6, 12, 13, 24, 34, 53, 56, 74, 77, 83^ One recent randomized controlled study^3^ found significant changes in fMRI of subjects with chronic back pain before and after psychological pain treatment, showing a possibility of using fMRI in pain measurement. However, fMRI is rather expensive and hard to do for routine use. Less expensive and easy-to-use devices that can also show changes that are consistent with chronic pain are needed.

This research^3^ along with another randomized controlled study^27^ demonstrated interventions that not only reduced but in many cases cured chronic pain by retraining the brain. Techniques for retraining the brain away from chronic pain include psychological therapies,^1,25,28,29,33,38,42,46,47,51,57,62,68–70,88,89,97^ education,^61,64,67,79,85^ biofeedback,^31,76,80^ activities,^2,17,44,54,55,63,87,91,92,96^ and meditation.^7,32,43,102^ Multimodal combinations of these techniques have been successful.^9,19,21,26,35,37,41,50,93^

The changes in the brain that can cause chronic pain may also cause physiological changes that can be measured. Recently, there has been significant progress in quantification of pain with physiological sensors.^10,14–16,20,30,39,45,58,60,72,73,78,86,98,100,101,103^ Sensors that measure physiological signals are usually low-cost and easy-to-use. By measuring physiological changes due to chronic pain, such a device could potentially be useful to diagnose chronic pain more accurately, which can in turn assist in pain reduction. Such a device does not directly help reducing the pain; instead, it assists in pain reduction by better assessing the pain. For example, one can use it to monitor pain before and after an intervention to assess the effectiveness of pain reducing techniques described above. Additionally, quantification of pain is important because there is increasing concern that gender, race, age, and intellectual development disabilities may be involved in diagnostic delays as well as overtreatment or undertreatment of pain.^8, 23, 52, 66^

A growing body of literature has suggested that self-reported acute pain and experimentally induced acute pain are associated with differences in physiological parameters—for example, heart rate and heart rate variability (HRV); blood pressure; peripheral pulsatile components of the cardiac cycle; and electrodermal activity.^20,30,45,78,100,101^ Much of this work is consistent with the notion that *multivariate assessments* are superior to single-parameter models when predicting the subjective experience of pain intensity. For example, in an experimental induction of acute thermal pain, *only a linear combination* of physiological data from electrocardiograms (ECG), photoplethysmograms (PPG), and galvanic skin response significantly differentiated between no-pain and low-, medium-, and high-pain states—*no individual parameter* was able to distinguish between no-pain and pain states alone,^86^ suggesting that information from multiple autonomic physiological signals may indeed offer the most promising avenue for objective pain quantification.^58^ This modeling approach extended to cases of pure nociception under anesthesia, where the conscious perception of pain was ostensibly impossible: a multivariate approach including HRV, PPG, and pulse transition time accounted for intra-operative clinical measures of noxious intensity of incision.^73^ Similarly, in another study, heart rate, HRV, PPG, galvanic skin response, and associated temporal/spectral subcomponents of those physiological signals differentiated between noxious and nonnoxious surgical events.^10^

Of special relevance to the present study, previous research^103^ has highlighted the promise of machine learning models for pain assessment. Multilayer artificial neural networks using features derived from skin conductance and ECG distinguished between no pain and moderate-to-high levels of experimentally induced thermal pain, with a combination of the 2 features outperforming either measure alone^60^; in the case of chronic pain resulting from sickle cell anemia, multinomial logistic regression using 6 physiological features significantly predicted pain scores on an 11-point pain scale—both within and between individuals.^98^ Thus, there is considerable evidence to suggest that multivariate assessments of classical physiological measures can inform our understanding of pain intensity. Critically, however, *there is no apparent consensus* on the optimal set of features to use: the pioneering work on this question has explored a highly diverse field of potential physiological parameters.^14–16,24,39,40,48,49,59,65,81,82,84,90,94,95,99^

In this study, we report preliminary pain prediction results using physiological data collected from chronic pain sufferers on our new Pain Meter. We investigated both individual-level models and an overall, population-level model, spanning various combinations of pulse and temperature features. At the individual-level model, our results show that the ability to quantify chronic pain can vary quite considerably, with only half of the participants showing moderately strong predictive accuracies. Aggregating the data into a population-level model, however, significantly predicted pain scores across each recording session, providing preliminary evidence for a generalizable model of chronic pain quantification using physiological parameters.

## 2. Methods

### 2.1. Pain meter

The prototype equipment was built to allow subjects to record at home. For safety, we avoided any sensors that required electrode contact to the subjects' bodies. These in-home subject recordings enable us to capture more natural variation in pain levels for each subject, including high pain levels that would have made travel to our laboratory difficult. The ability to create subject-specific chronic pain models is due to the ability to record in homes.

**Figure 1.**
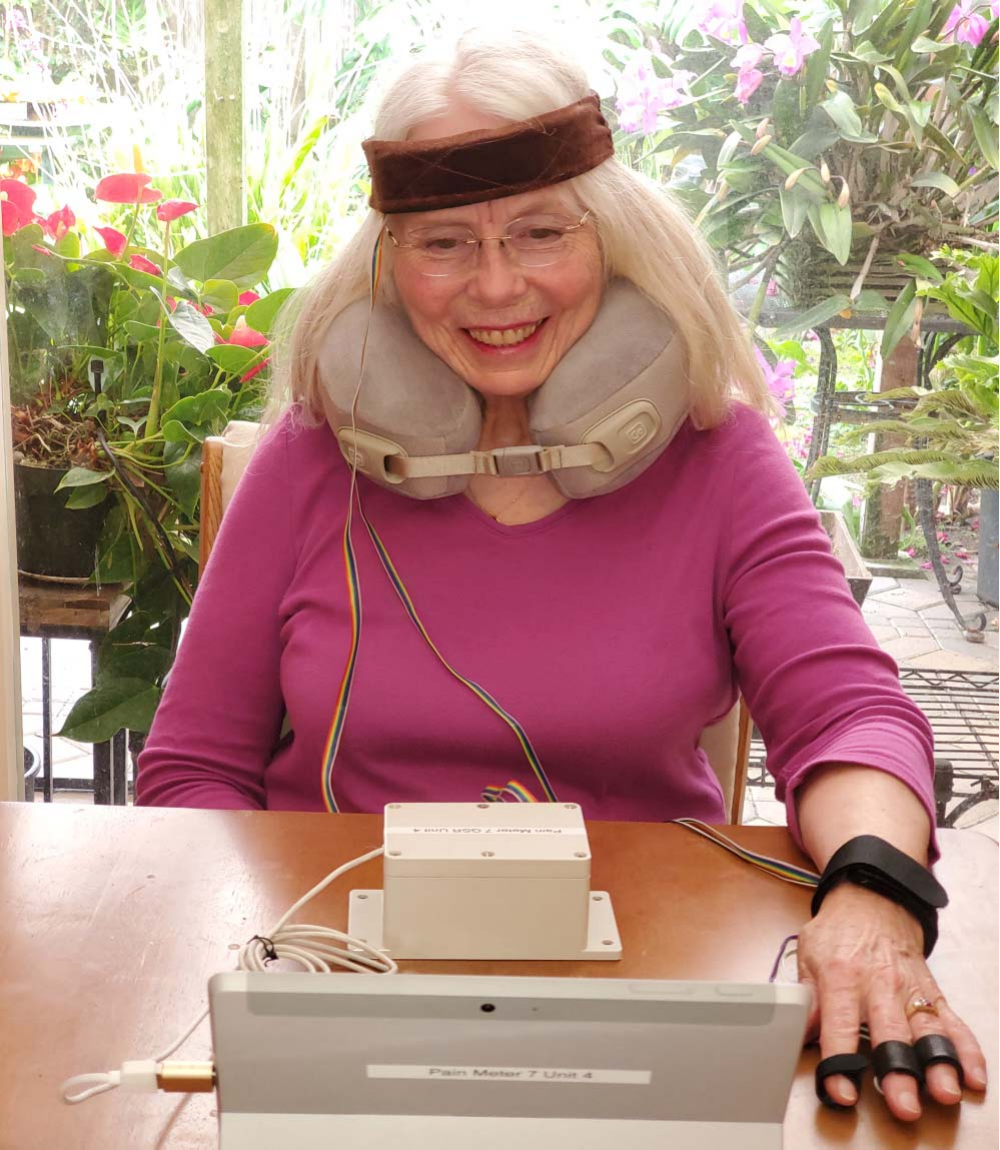
Pain Meter contains (1) PPG pulse sensors in a headband, in a neck pillow, wristband, and on the fingertip; (2) temperature sensors on the temple, forehead, wrist, and fingertip; (3) 3-axis accelerometers and 3-axis gyros in the headband and wristband; and (4) force sensors on the left carotid and basilar. PPG, photoplethysmograms.

**Figure 2.**
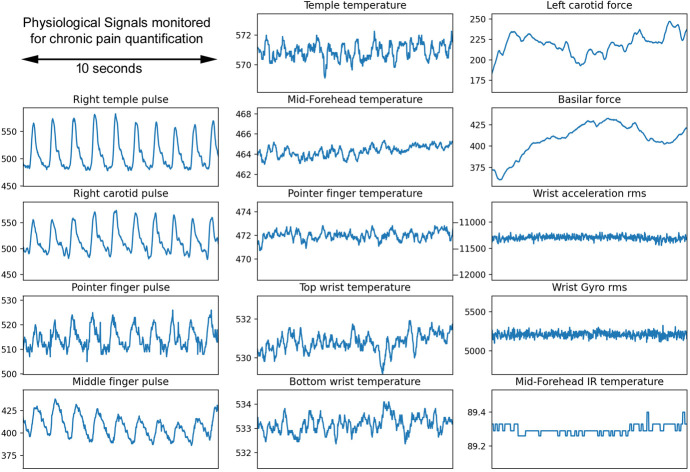
An example of the signals recorded from Pain Meter.

Our Prototype Pain Meter (Fig. 1) represents the seventh iteration of our device. The sensors we chose were inexpensive and available commercially: photoplethysmograms pulse sensors (Pulse Sensor Amplified; http://www.pulsesensor.com); temperature sensors (10K Ohm NTC Thermistors MF52-103); IR temperature sensor (MLX 90614); acceleration/gyroscope sensors (HiLetgo GY-521 MPU-6050 MPU6050 3 Axis Accelerometer Gyroscope Modules); and force sensors (Interlink FSR 402 on the forehead and wrist; and Interlink 406 on the basilar and carotid arteries). An example of the signals recorded from Pain Meter is shown in Figure 2. The Prototype Pain Meter does not apply stimulation to obtain different pain score recordings.

A Teensy 3.6 microcontroller with a 32-bit 180 MHz ARM Cortex-M4 processor was used to sample all signals at a frequency of 66.67 Hz. Data visualization and acquisition programs for our Pain Meter were written in Python. Data were remotely monitored using the Dropbox cloud storage system.

### 2.2. Participants

Data were collected from 12 participants (9 women and 3 men; *M*_age_ = 38.25, *SD* = 17.10, range = 21–65), yielding a total of 183 10-minute recordings. Importantly, we did not specify any explicit exclusion criteria for participation: thus, pain etiology/symptomatology, duration, and intensity varied across individuals. For transparency, we summarize these factors in Supplementary Table 1 (available at http://links.lww.com/PR9/A173).

All subjects were recruited via email and provided written informed consent for a protocol approved by the UCSB Human Subjects Committee.

### 2.3. Data collection

Subjects first opened a computer program on the Pain Meter computer that was supplied to them. They connected to Wi-Fi on the first use. Then they were asked to rate the best representation of their current pain score using a visual analogue scale, which included pictures and short descriptions of pain states from 0 to 10 (eg, the description of state 0 was “no pain” and the description of state 10 was “unimaginable unspeakable”).

Subjects next put on the headband and neck pillow sensors, adjusted their position until they felt comfortable, and secured the sensors with Velcro (headband) and a fastener (neck pillow). Next, they put on a wrist band and 2 finger cuff sensors. Once these were secured, the computer monitored all the pulse signals and gave green lights for all sensors giving good signals (signals with clearly detected peaks) and yellow for signals that were not. If there were any yellow lights, the subject was asked to readjust the sensor and try again.

Once all the sensors were giving good signals, a 10-minute recording session was initiated in which pulse, temperature, force, and motion data were recorded and stored in local memory (at the end of the session, the complete record was automatically uploaded to the cloud).

During this recording, the subject was instructed to relax and to look at a fixation cross on the computer screen. The data being recorded was not visible to the subjects during the recording. After the recording was done, the subjects were asked to report their current pain score again with the same visual analogue scale. The protocol instructed subjects to record for 2 weeks, twice per day.

### 2.4. Data preprocessing

**Figure 3.**
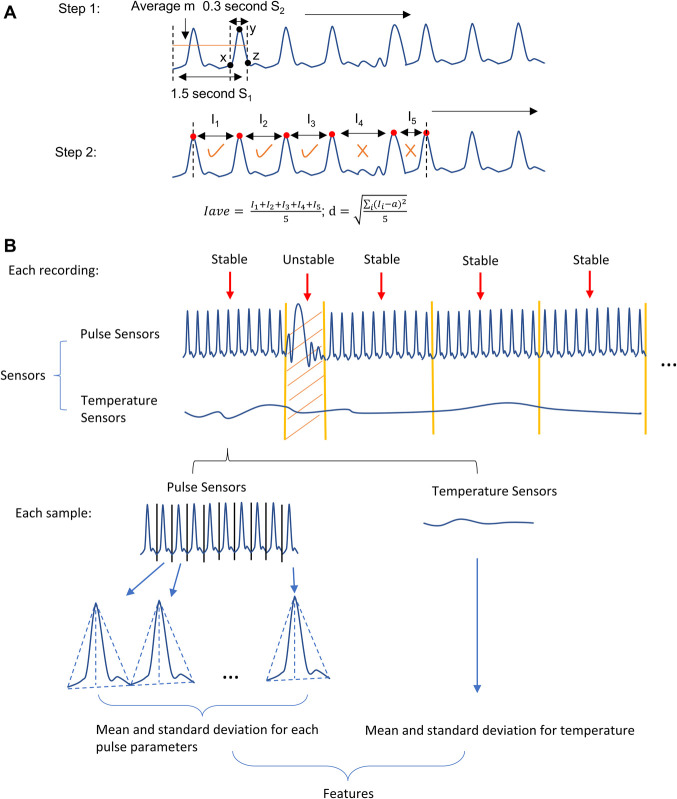
Preprocessing and feature extraction. (A) Step 1: Detect all pulse peaks in the recording by comparing the moving average *m* of each 100 data-point segment in S1 (S1 is 1.5-second-long) with the last 20 data points in S2 (S2 is 0.3-second-long) of S1. If the first and last data point in S2 is smaller than *m* and the max value of S2 is larger than *m*, then a peak is detected. Step 2: Take the first 6 peaks and calculate the mean *I*_ave_ and SD *d* of the time between peaks. If *I*_ave_ is between 0.6 and 1.2 seconds and *d* is smaller than 0.2 and if the time *I* between 2 peaks is between 0.9 and 1.1 times I_ave_, this data segment is classified as stable. Otherwise, this data segment is classified as unstable. Repeat this process for all the data. (B) The data preprocessing and feature extraction steps: (1) Remove unstable segments and divide the rest of the recording into continuous 10-second samples. (2) Inside each sample, extract pulse features such as the mean and SD for each pulse parameters and mean and SD for temperature.

Movements of the subjects during the recording sessions can disturb the pulse signal and add additional noise into the data. Consequently, it can be difficult to extract robust, interpretable features from individual pulses. We use several steps to preprocess the data and reduce the influence of noise in our analysis. After unstable segments are detected (as detailed in Fig. 3), we remove them and split the stable segments into 10-second samples before proceeding to feature extraction. We used 3 pulse sensors in our analysis: temple pulse sensor, carotid pulse sensor, and pointer finger pulse sensor. Note that, because we removed all unstable segments, the number of available recordings and samples differed depending on the combination of sensors—Table 1 shows the number of stable recordings and samples for each combination.

**Table 1:** Details of the models trained with different combination of pulse sensors.

Pulse sensors	# of recordings	# of samples	*r*	RMSE
1	173	7755	0.60	1.52
2	168	6671	0.44	1.78
3	179	8900	0.60	1.52
1, 2	156	5446	0.45	1.69
1, 3	168	7033	0.57	1.56
2, 3	163	5978	0.49	1.66
1, 2, 3	152	5044	0.41	1.71

Number of recordings, number of samples, Pearson correlation coefficient (*r*), and root mean square error (RMSE) are shown. For pulse sensors, 1 represents temple pulse sensor; 2 represents carotid pulse sensor; and 3 represents pointer finger pulse sensor.

Participants provided one pain score at the beginning of each recording and then again at the conclusion. We did not give any instructions to the participants such as telling them to relax or in any way attempt to change their pain. Nevertheless, participants often reported a different pain level at the end of the session. We therefore split the data and assigned the first pain score to samples from the first half of each recording and the second pain score to samples from the second half. This is, of course, only an approximation because we have no information about when the pain level changed, but it seemed preferable to using only the beginning pain score or only the end pain score for the entire recording.

### 2.5. Feature extraction

Each 10-second sample included stable signals from the pulse sensors and temperature sensors. We defined a series of pulse features analogously to earlier research,^44^ which suggested that pulse morphology derived from transmitted-light PPG can be used for postoperative pain assessment. Our device, however, uses green reflective-light pulse sensors with highly filtered output (because they are both inexpensive and readily available). We therefore investigated whether our pulse sensor parameters can inform the quantification of chronic pain.

**Figure 4.**
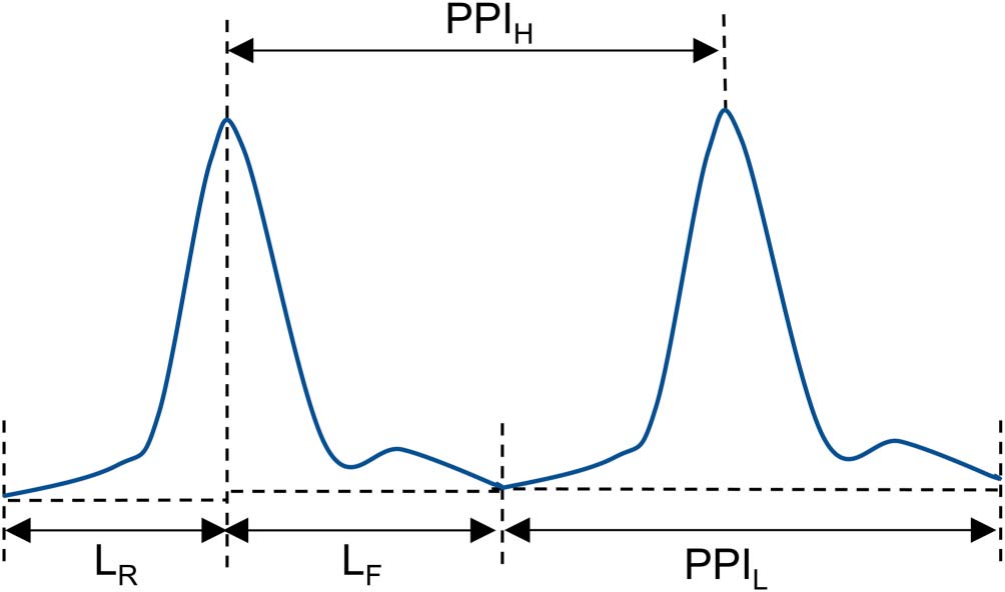
Pulse parameters. L_R_ is the width of the rising part of the pulse; L_F_ is the width of the falling part of the pulse; PPI_H_ is the interval between 2 consecutive highs; and PPI_L_ is the interval between 2 consecutive lows.

We extracted only pulse parameters that are independent of the height of the pulse because the height of the pulse can be influenced by how tightly the device is worn and its position. Specifically, we extracted pulse width parameters: L_R_, L_F_, PPI_H_, and PPI_L_ (Fig. 4). PPI_H_ is defined as the interval between 2 consecutive high peaks of pulses. PPI_L_ is defined as the interval between 2 consecutive low peaks of pulses. The SD of PPI_H_ is a measure of pulse rate variability, which was used as one of the parameters in our models.

From each 10-second sample, after finding the peaks, we segmented the data into individual pulses by finding the local minimum between peaks. Figure 3B illustrates this process.

Thus, the following features were extracted from the samples:

(1) Mean and SD of all pulse sensor parameters.
(2) Mean of temperature at forehead, temple, top of wrist, and bottom of wrist and finger.

Additionally, we computed $\bar{T_{Temple}}$ / $\bar{T_{Finger}}$, $\bar{T_{Temple}}$ - $\bar{T_{Finger}}$, $\bar{T_{Forehead}}$ / $\bar{T_{Finger}}$, $\bar{T_{Forehead}}$ - $\bar{T_{Finger}}$, where $\bar{T_{Temple}}$ represents the temple temperature mean for each 10-second sample window, $\bar{T_{Forehead}}$ represents the forehead temperature mean and $\bar{T_{Finger}}$, represents the finger temperature mean. All features were z-scored normalized before further analysis.

### 2.6. Recursive Feature elimination and random forest regression

Feature selection methods are used to identify a subset of “important” features and simplify a model. These techniques often afford several advantages, such as reducing computational costs, avoiding overfitting, and improving model performance.

One popular method, Recursive Feature Elimination, selects features by training a model with one set of features, ranking them by an importance metric, and removing the least important. This procedure is recursively repeated until the user-specified number of features is reached. We implemented subject-specific linear models for individualized pain prediction, accounting for the fact that relevant physiological parameters could likely vary on a subject-by-subject basis.

At the overall population level, we used random forest regression: an ensemble method that aggregates the predictions from multiple regression trees to make more accurate predictions. Regression trees are decision trees when the outcomes are continuous values, recursively partitioning the data space into smaller regions where simple models can be used to describe the relationships between various model inputs and outcomes. In random forest, each regression tree is created using random subsets of features and random subsets of samples. Accordingly, because random forest uses different subsets from the data and features, it is less likely to overfit and performs well with high-dimensional datasets. Additional advantages include fewer constraints and assumptions about the data and its distribution, as well as robustness to outliers and missing values. After the forest is fit, the predicted pain score of the test set is computed as the mean predicted score of the trees in the forest.

This part of the analysis was done with the Python package *scikit-learn*.^71^

### 2.7. Leave-one-recording-out cross-validation

We used leave-one-recording-out cross-validation to assess performance in all models (individual level and population level). For leave-one-recording-out cross-validation, all samples from one recording were iteratively removed from the dataset, the model was trained on the remaining data, and predicted pain scores were obtained for the left-out recording. Pearson correlation coefficient (r), intraclass correlation coefficient, and root mean square error (RMSE) between the predicted pain ratings and the reported values are reported. Intraclass correlation coefficients (ICC(3, 1)) are calculated with R package *psych*.^89^

We additionally performed 1000 iterations of nonparametric permutation tests to assess model performance against chance. In this procedure, we constructed a robust empirical null distribution of predictive accuracies expected if pain scores were randomly associated with patterns of physiological parameters. Thus, for each permutation, pain scores were shuffled randomly for all recordings, models were fit using cross-validation as above, and performance was recorded. Significance was determined by the proportion of cases in which null-model accuracy equalled or exceeded the “true” accuracy.

Because Pearson correlation is not an agreement statistic, a high correlation does not necessarily imply a good agreement between the 2 scores. Thus, we also ran a Bland–Altman analysis for our population-level model.

Despite these checks, there are still limitations and potential overfit problems with our methods. Future studies are needed to validate stable models against independent subject samples.

## 3. Results

We were able to capture a fairly wide range of pain scores (spanning 0–9) through our home recording sessions. However, we note that more extreme ratings (particularly, pain scores 0, 8, and 9) have many fewer samples for analysis relative to the other values. We elected to retain these recordings given the continuous nature of our regression frameworks, but the imbalanced representation of scale endpoints is worth keeping in mind for model assessment.

### 3.1. Individual-level models

**Figure 5.**
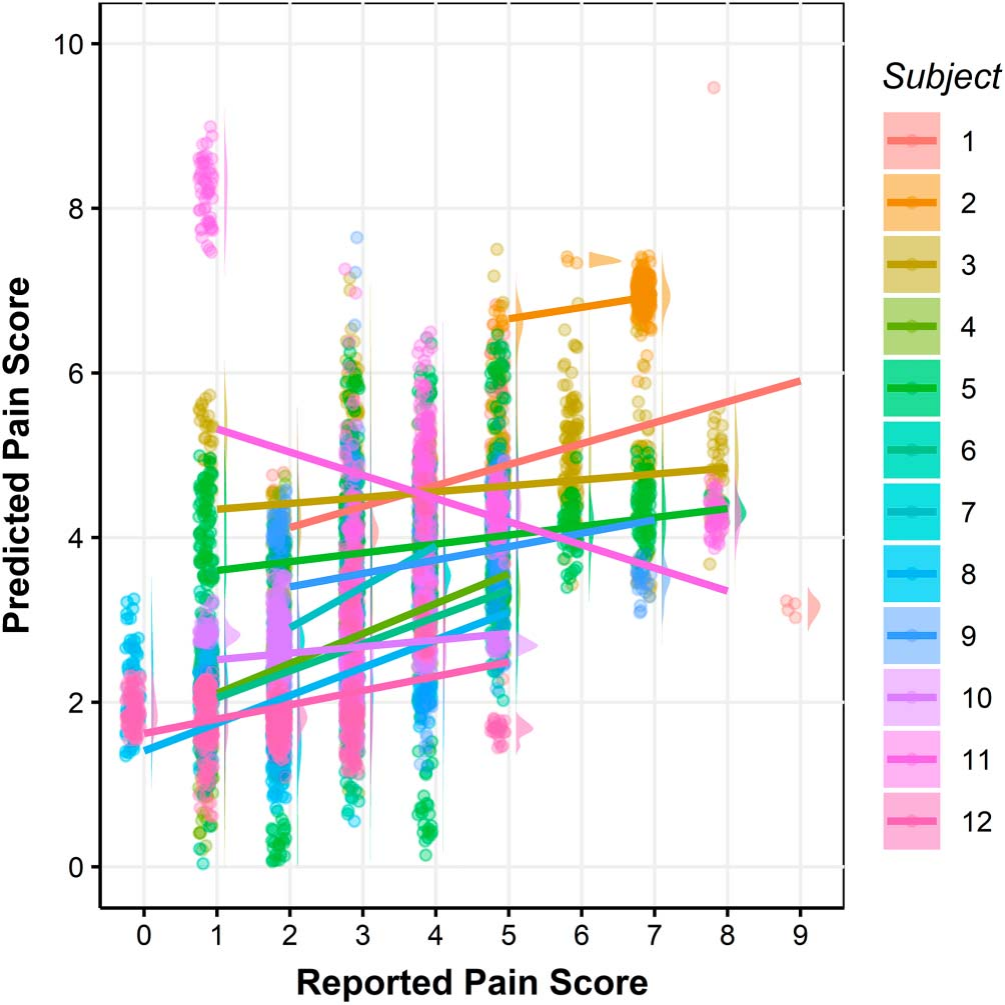
Cross-validated predicted pain scores vs reported pain scores for each subject. Different colors represent different subjects in the raincloud plots. Each dot represents one sample, and each line is the fitted linear regression line for each subject. In total, there are 6982 samples, 99.50% of the data is shown in the figure. The rest were omitted to provide higher resolution for the bulk of the data. The Pearson correlation coefficients between reported and the predicted pain scores are 0.78, 0.29, 0.14, 0.54, 0.19, 0.49, 0.56, 0.50, 0.24, 0.16, −0.31, and 0.35, respectively, for subjects 1 through 12.

We first trained and cross-validated predictive models on an individualized, within-subject basis. Because of the smaller sample size for individual subjects, we used recursive feature elimination to select 5 features first to avoid overfitting and then fit a linear model to predict the pain scores for each subject. All combinations of pulse sensors were tested for each subject. The models with the combination that gave the best predictive performance (ie, correlations between reported and predicted pain) are reported in Figure 5. As expected, performance was rather variable across individuals. However, pain scores in 5 of 12 subjects were predicted with moderately strong accuracy (0.49 < *r* < 0.78, *P* < 0.05). The intraclass correlation coefficients for these 5 subjects are between 0.46 and 0.75. A few features were selected by recursive feature elimination repeatedly in these 5 models. For example, $\bar{PPI_{L}}$ was selected in 5 of 5 models and $\bar{L_{F}\;}$, $\bar{T_{Forehead}},\;\bar{T_{Finger}}$ and $\bar{T_{Forehead}}$ - $\bar{T_{Finger}}$ were selected in 4 of 5 models. However, the relationships between these features with pain score are not consistent across different individuals. For example, $\bar{T_{Forehead}}$ and $\bar{T_{Finger}}$ increased with the increase of pain score in 2 of 4 subjects and decreased in the other 2 subjects.

### 3.2. Population-level model

We then aggregated the data from all subjects and fit population-level random forest models using different combinations of pulse sensors. Table 1 shows the results from each possible combination of sensors. In general, modeling more pulse sensors simultaneously leads to a smaller number of potential training samples overall (because a given 10-second window was only used if it was stable for *all* sensors combined). Models including *only* the pointer finger sensor or *only* the temple sensor gave the strongest correspondences between actual and predicted pain (*r* = 0.60, *RMSE* = 1.52). The intraclass correlation coefficient was 0.58 for both models, showing a moderate similarity between actual and predicted pain.

**Figure 6.**
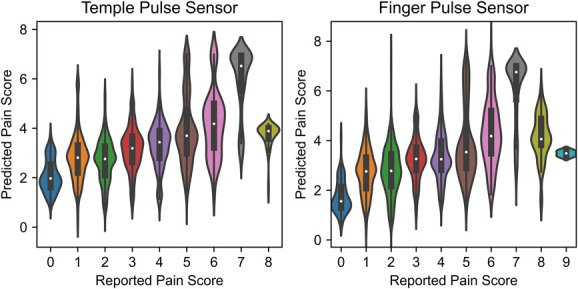
Cross-validated predicted pain scores vs reported pain scores for population-level models. Left panel shows the results from the model using signals from temple pulse sensors, and right panel shows the results from the model using signals from pointer finger pulse sensors. The violin plots for each class shows the distributions of the predicted pain scores.

However, as aforementioned, predictive accuracy generally suffered the worst at pain scores with fewer available examples (Fig. 6). Because the number of stable recordings and samples varies when using different pulse sensors, there are different number of samples for each pain score. (The number of samples for each pain score are shown in Supplementary Table 2, available at http://links.lww.com/PR9/A173.) The more extreme ends of pain score (8 and 9), in particular, were considerably undershot by the model. Nonetheless, performance on the whole consistently outperformed empirical null models, suggesting that pain prediction was still significantly better than chance (*P* < 0.001).

Bland–Altman plots for both models are shown in Supplementary Figure 1 (available at http://links.lww.com/PR9/A173). Because one of the measurements in this article is the self-reported pain, which we consider as our reference measurement, we used the self-reported pain scores as the *x*-axis in our Bland–Altman plots. Note that the self-reported pain scores are integers from 0 to 10; thus, all dots in the plots lie on vertical lines with integer x value. The bias and agreement limits are the same for both models. The bias is 0.02, and the range of agreements is −2.95 to 2.99. Our model tends to overestimate the lower end of the pain scores and underestimate the higher end of the pain scores. Note that plotting against reference measurement instead of average measurements might be responsible for the association between difference and pain scores.^11^

## 4. Discussion

This is the first demonstration, to our knowledge, that physiological data can be correlated with chronic pain, both for individuals and populations. In our subject-specific models for individualized pain prediction, 5 of 12 subjects yielded Pearson correlation coefficients of 0.49 (*P* < 0.05) to 0.78 (*P* < 0.05) and intraclass correlation coefficients between 0.46 and 0.75. In our population-level model, we achieved an intraclass correlation coefficient of 0.58 and a significant Pearson correlation between self-reported and predicted pain (*r* = 0.60, *P* < 0.001) in this preliminary study. It is likely that higher correlations will be achieved with better devices in the future. This first demonstration had significant limitations due to the limited amount of data that we could collect shortly before and during COVID-19. Additional research is urgently needed to explore the effects of the type and duration of chronic pain, the age and gender of the subjects, and other factors.

Additional research is also needed to optimize the selection and placement of sensors. As one example of possible future improvements, an optimized pulse sensor would be able to sense the low-frequency variations in the baseline due to breathing, which were filtered out in the commercial pulse sensor that we used. It is also possible (perhaps even probable) that the inclusion of ECG, electromyography, or skin impedance sensors would give better correlations. However, in the interest of subject safety, because these were unsupervised measurements in the subjects' own homes, we did not include any devices with electrodes.

Our long-term goal is the development of a relatively low-cost, easy-to-use system that could be used by patients in their own homes or in physician offices to objectively measure chronic pain. Hopefully, this first demonstration of the possibility of achieving this goal will inspire engineers, physicists, computer scientists, and psychologists to join in doing the work that will be necessary to move from the demonstration of a possibility to a practical system. The current system of subjective self-reported pain levels results in the overprescribing and underprescribing of treatment. Shifting to an objective methodology would provide health care professionals the ability to properly prescribe patients with the appropriate dosage of treatment to alleviate pain. This is especially important with the many risks associated with pain medication, including long-term opioid addiction.

## Disclosures

The authors have no conflicts of interest to declare.

## Appendix A. Supplemental digital content

Supplemental digital content associated with this article can be found online at http://links.lww.com/PR9/A173.

## Supplementary Material

SUPPLEMENTARY MATERIAL
