# Supplementary material for: Preliminary study: quantification of chronic pain from physiological data
Source: Pain Rep. 2022 Oct 4;7(6):e1039. doi: 10.1097/PR9.0000000000001039 (PMC9534370; doi:10.1097/PR9.0000000000001039)

Supplementary Table 1. Subject information.

ID	Gender	Age	Race	description of pain
1	F	30	White	Primarily lower back pain
2	M	65	White	Cervical, lumbar, hand pain and numbness
3	F	58	White	Fibromyalgia. Highly variable per location, weather, and activities. Sometimes sharp, sometimes achy.
4	F	60	White	Upper back pain
5	F	27	Asian	Back of neck, between should blades in upper/mid-back and lower back, jaw, hips, possible fibromyalgia
6	F	29	White	Neck, upper back, behind eyes, fibromyalgia and chronic migraines
7	F	48	White	Fibromyalgia pain in right thumb and middle finger, left elbow and tip of both shoulders, also back pain and knee pain
8	F	19	White & Asian	Lower back, usually an aching pain but occasionally a sharp pain that causes left leg to buckle.
9	M	21	White	Fibromyalgia, upper/lower back, chest, most joints
10	F	53	White & Asian Pacific Islander	Pain from Endometriosis, Adenomyosis, Adhesions / Surgical Scars, abdomen, lower back, pelvic floor, hips, legs, torso
11	F	24	White	Migraines
12	M	25	White	Fibromyalgia global

ID	Duration of chronic pain	Max pain in a typical week	Average pain in a typical week	Min pain in a typical week	Medication
1	7 y	9	4	1	Not Provided
2	35 y cervical, 50 y lumbar, 2 y hand	8~9	7~8	7	Not Provided
3	19 y	8	6	4~5	Not Provided
4	1 y	Not Provided	Not Provided	Not Provided	Not Provided
5	2.5 y	10	5~6	1~2	Aleve, Excedrin, Amitryptylin
6	17 y	8	5.5	4	Not Provided
7	14 y	Fibro: 8, back: 9, knee: 10	Fibro: 4, back: 5, knee: 8	Fibro: 1, back: 1, knee: 6	Meloxicam
8	3 y	8	3	1	Advil
9	3-4 y	8	6	3	Gabapentin, Cymbalta, Ibuprofen, Tylenol, Cyclobenzaprine
10	30+ y	8	6	3	Ibuprofen, RX Tens Machine
11	9 y	10	4~6	2	Aimovig 140mg, Nurtec 75mg
12	5 y	5	3	2	None

Supplementary Table 2. Number of stable recordings and samples with temple pulse sensor or finger pulse sensor.

Temple Pulse Sensor:

Pain Level	0	1	2	3	4	5	6	7	8	9
# of Recordings	8	37	61	76	97	47	11	47	3	0
# of Samples	206	956	1507	1770	1863	908	226	797	73	0

Finger Pulse Sensor:

Pain Level	0	1	2	3	4	5	6	7	8	9
# of Recordings	8	36	63	78	64	42	9	47	6	2
# of Samples	232	1020	1671	2096	2311	1080	208	857	154	4

Supplementary Figure 1. Bland Altman Plots. Left panel shows the results from the model using signals from temple pulse sensors and right panel shows the results from the model using signals from pointer finger pulse sensors.

Temple Pulse Sensor

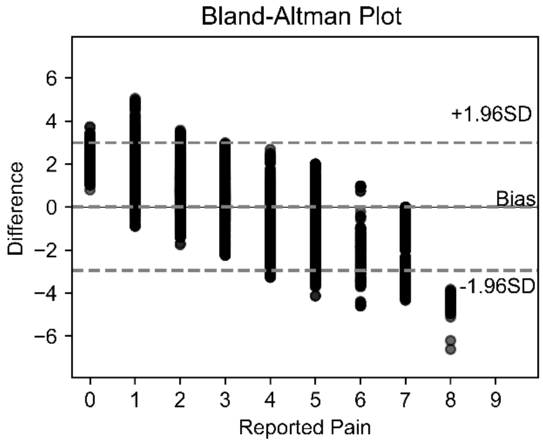

Finger Pulse Sensor

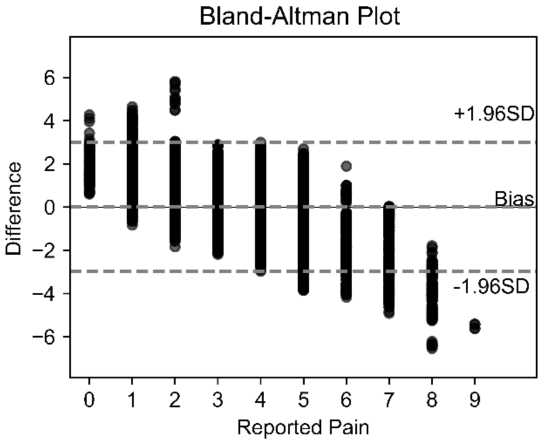

Supplement: SUPPLEMENTARY MATERIAL [file painreports-7-e1039-s001.pdf]
